# Challenging diagnosis of *Plasmodium ovale* malaria in a Colombian traveler: the importance of including *P. ovale wallikeri* in molecular screening

**DOI:** 10.1590/S1678-9946202466029

**Published:** 2024-05-13

**Authors:** Carlos Nieto-Clavijo, Liliana Morales, Angela Patricia Guerra Vega, Liliana Jazmín Cortés Cortés, Jacqueline Chaparro-Olaya

**Affiliations:** 1 Universidad El Bosque, Vicerrectoría de Investigaciones, Laboratorio de Parasitología Molecular, Bogotá, Colombia; 2 Instituto Nacional de Salud, Dirección de Redes en Salud Pública, Grupo de Parasitología, Bogotá, Colombia

**Keywords:** Colombia, Plasmodium ovale, Diagnostic errors, Travel-related illness

## Abstract

This study reports a challenging diagnosis of *Plasmodium ovale* malaria in a Colombian citizen returning from Cameroon. Initial microscopy screenings conducted at two private hospitals yielded conflicting results, with the first showing negative smears and the second diagnosing *P. vivax*. Subsequent microscopy examinations at two government laboratories identified *P. ovale*, although the routine species-specific PCR strategy was negative. PCR confirmation was finally obtained when *P. ovale wallikeri* primers were used. Although *P. ovale* is not frequently found in Colombia, there is a clear need to include both *P. ovale curtisi* and *P. ovale wallikeri* in the molecular diagnostic strategy. Such need stems primarily from their extended latency period, which affects travelers, the increasing number of African migrants, and the importance of accurately mapping the distribution of *Plasmodium* species in Colombia.

## INTRODUCTION

In 2021, an estimated 247 million malaria cases and 619,000 malaria-related deaths were reported in 84 malaria-endemic countries. WHO African Region accounted for approximately 95% of global cases, with an estimated 234 million cases in 2021. In the WHO Region of the Americas, Venezuela, Brazil, and Colombia accounted for more than 79% of all cases^1^.

*Plasmodium ovale* is one of the five *Plasmodium* species capable of infecting humans. It is most prevalent in tropical West Africa and the Southwestern Pacific; outside these regions, it is extremely rare (1%). Currently, information on malaria caused by *P. ovale* is scarce. A search for “*Plasmodium ovale*” and “Colombia” on PubMed, Embase, and LILACS (on the November 1^st^ 2023) yielded a single specific record corresponding to the first report of *P. ovale* malaria in the country^2^. Due to the morphological similarity between *P. vivax* and *P. ovale*, differentiating these two species can be challenging, even for a well-trained or expert microscopist. In fact, a recent meta-analysis revealed that misidentification of *P. ovale* as *P. vivax* by microscopy can occur in up to 11% of cases^3^. The increasing prevalence of *P. ovale* observed worldwide and the increasing number of African migrants crossing the Colombian territory to seek asylum in the United States highlight the importance of implementing highly responsive surveillance to accurately identify this species.

## CASE REPORT

Written informed consent was obtained from the patient for publication of this case report. A 29-year-old Colombian man was admitted to a second-level private hospital in Bogota, Colombia. The patient presented a 10-day history of unspecified recurrent fever (39 °C) accompanied by nocturnal diaphoresis, shortness of breath and muscle weakness. He mentioned that he had returned from Douala, Cameroon (Africa), 10 days earlier and that three of his traveling companions had tested positive for malaria by thick blood smear. The patient had visited another private hospital several days earlier, where he had a normal hemogram, two negative malaria thick blood smears, and a negative test for dengue antibodies. A 1g metamizole was administered and the patient was then discharged with a paracetamol prescription.

On admission to the second hospital, the patient was alert, afebrile (37.1 °C), with a blood pressure of 110/62 mmHg, an elevated heart rate (129/min), normal oxygen saturation (96%), normal respiratory rate (20/min), normal posture, and a normal neurological exam (Glasgow score 15/15). Laboratory tests were ordered. Blood tests revealed leukopenia (WBC count of 3,650/µL with 81.4% neutrophils and 11.8% lymphocytes), thrombocytopenia (76,000 platelets/µL), normal hematocrit (42.8%), and normal hemoglobin concentration (15.4 g/dL). The total, direct and indirect bilirubin levels were 0.67, 0.24 and 0.43 mg/dL, respectively. Creatinine was 0.62 mg/dL and liver enzymes levels were 19 U/L of aspartate aminotransferase (AST) and 20 U/L of Glutamic Pyruvic Transaminase (GPT). Lastly, the thick blood smear was positive for *P. vivax* (2,190/μL parasite count) and revealed the presence of gametocytes. The infection was treated with the established regimen for uncomplicated malaria caused by *P. vivax*: Day 1: 600 mg of chloroquine and 15 mg of primaquine; days 2 and 3: 450mg of chloroquine and 15 mg of primaquine; days 4 to 14: 15 mg of primaquine. The patient remained hospitalized for one day, was referred for consultation with an infectious disease specialist, and was eventually discharged after a normal hepatobiliary ultrasound, improvement of symptoms, and absence of vomiting episodes.

An EDTA-anticoagulated blood sample, along with thick and thin blood smears, was sent to the Public Health Laboratory (LSP) of the Bogota District Health Department, where *P. ovale* infection was diagnosed by microscopy. The LSP then sent the sample and smears to the Colombian National Institute of Health (INS) for species confirmation by PCR.

**Figure 1:**
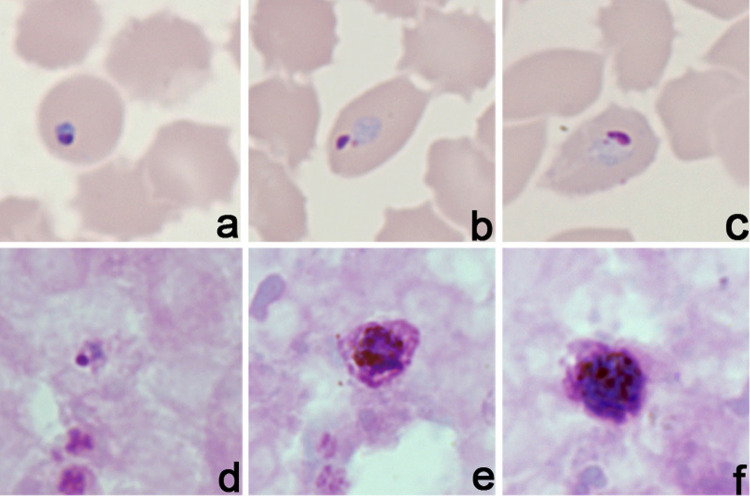
Microphotographs of *Plasmodium ovale* in field-stained thin and thick blood smears. Magnification 1000X showing thin (a-c) and thick (d-f) blood smears obtained from the patient under study: (a) ring; (b,c,d) young trophozoite; (e) late trophozoite; (f) gametocyte.

**Figure 2:**
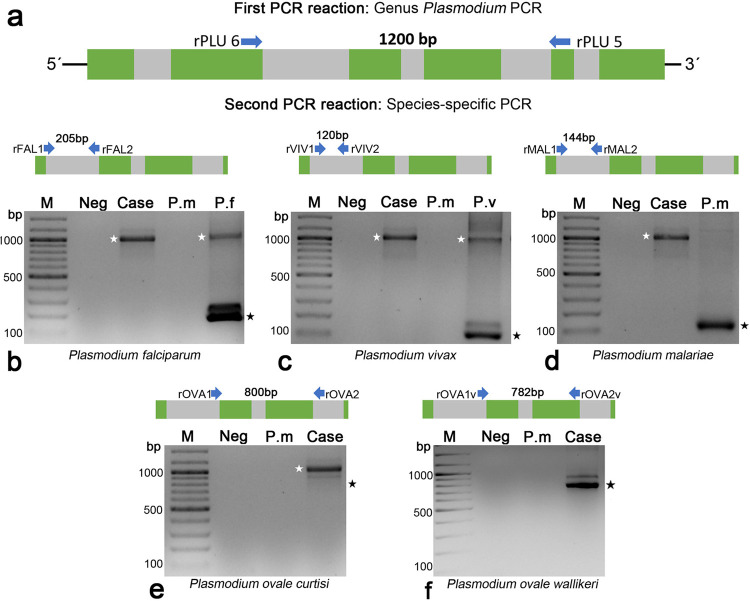
Species-specific PCR for *Plasmodium*: (a) PCR strategy; (b-f) agarose gels (2%) stained with ethidium bromide. PCR reaction with primers specific for *P. falciparum* (b), *P. vivax* (c), *P. malariae* (d), *P. ovale curtisi* (e), and *P. ovale wallikeri* (f). DNA from *P. falciparum* (P. f), *P. vivax* (P. v) and *P. malariae* (P. m) was used as a control in the PCR assays. Neg = Negative control (reaction without template); Case = DNA from the blood sample of the patient under study; M = Molecular weight marker (GeneRuler 100 bp Plus DNA Ladder. Thermo). The white stars indicate the PCR product obtained from the first PCR test (this 1200 pb amplicon is shared by *Plasmodium* sp. and was used as a template in the species-specific PCR). The black stars indicate the expected size for species-specific PCR products.

The presence of *P. ovale* infection was confirmed at the INS by microscopy (Figure 1), and the anticoagulated blood sample was then used for DNA extraction using the QIAamp^®^ DNA Micro Kit (Qiagen, Hilden, Germany). Molecular diagnosis of *Plasmodium* species was obtained using the strategy described by Snounou *et al*. in 1993^4^. This PCR-based approach involves a first amplification with primers targeting a DNA sequence shared by *Plasmodium* species on their small subunit ribosomal RNA (ssrRNA) (primers rPLU6 and rPLU5). A second PCR reaction is then performed with primers unique to *P. falciparum* (rFAL1 and rFAL2), *P. vivax* (rVIV1 and rVIV2), *P. malariae* (rMAL1 and rMAL2), or *P. ovale* (rOVA1 and rOVA2) (Figure 2a). PCR was performed according to the conditions described by Cortés and Guerra in 2020^5^. Although the patient’s sample was positive in the first reaction, indicating the presence of *Plasmodium*, no amplification for any of the four species was obtained in the second PCR (Figure 2b-2e). In fact, when the species-specific PCR reactions were subjected to electrophoresis, only a single product of approximately 1200 bp was observed. This product corresponded to the amplicon synthesized in the first PCR reaction, which served as template in the species-specific PCR. Since variations in the ssrRNA gene have been identified in *P. ovale* ^6^, different primers have been designed to detect the presence of these polymorphisms, which are now recognized as two different species: *P. ovale curtisi* (classical type) and *P. ovale wallikeri* (variant type)^7^. Therefore, an aliquot of the extracted DNA was sent to the Universidad El Bosque, where a PCR assay for *P. ovale wallikeri* was performed as described by Calderaro *et al*.^8^, using primers rOVA1v (5’-ATCTCCTTTACTTTTTTTTTTTTTGTACTGAGA-3’) and rOVA2v (5’- GGAAAAGGACACTATAATGTATTATCCTAATA-3’). The PCR product, an amplicon of approximately 800 bp, consistent with the expected size for the variant type (Figure 2f), was sequenced in both directions by Sanger sequencing (Macrogen, Korea). Subsequently, a local alignment search was conducted on the obtained sequence (GenBank accession OR605740) using the BLASTn tool, revealing 99% homology with *P. ovale wallikeri* sequences.

## DISCUSSION

In this study, we report the diagnostic path of a Colombian citizen returning from Africa. Malaria was initially ruled out and subsequently misdiagnosed as *P. vivax* malaria, until *P. ovale wallikeri* infection was finally confirmed. Although misidentification of *P. ovale* as *P. vivax* is not a clinical problem (as the same antimalarial treatment is administered), frequent misdiagnosis could misrepresent the distribution of *Plasmodium* species in the country.

Several studies have shown that imported *P. ovale* malaria primarily originates from Africa. In Southeastern China and Sweden, 98.5% and 95.2% of imported *P. ovale* cases were from Africa, respectively^9,10^. At the same time, although there are few reports of *P. ovale* as the main cause of severe malaria, a study on travelers and migrants conducted in Sweden over two decades showed that 5.3% of *P. ovale* infections resulted in severe episodes^10^. Thus, clinicians and technicians must be aware that patients returning from endemic countries may develop severe *P. ovale* infections. In addition, since 2013, an increasing number of African migrants have been crossing South and Central American countries to reach the US-Mexico border. African migrants typically arrive in Brazil, the most accessible Latin American country for Africans, continue to Peru, cross Ecuador to the Ecuador-Colombia border, and cross Colombia from south to north until they reach the border with Panama. The transit across Colombia covers more than 1,000 km^11^.

In addition, given the biology of *P. ovale curtisi* and *P. ovale walikeri* in the hypnozoite stage (as well as *P. vivax*), these parasites should not be neglected in malaria control programs. It is essential to detect infections or co-infections with *P. ovale*, as in these cases the administration of primaquine is mandatory to treat relapsing disease (hepatic hypnozoite stage). However, it is noteworthy that *P. ovale wallikeri* species have not been included in all PCR-based diagnostic assays.

Lastly, it is worth mentioning that some studies indicate significant differences in several aspects between *P. ovale curtisi* and *P. ovale wallikeri*, although with conflicting results. The only observations consistently reported in several studies are significantly higher thrombocytopenia^12-14^ and a shorter latency in patients with *P. ovale wallikeri* compared to those infected with *P. ovale curtisi* ^14-16^. In these studies, the median latency periods for *P. ovale curtisi* were 85.7 days (n = 74), 86 days (n=112), and 72 days (n=309), compared with 40.6 days (n = 60), 29 days (n=131), and 34 days (n=368) for *P. ovale wallikeri*. The longest latency period found in these studies was 1,083 days, highlighting the need to consider suspicious symptoms in any history of travel to endemic countries dating back months or even years. Failure to associate symptoms with travel can lead to delayed or missed diagnoses. Clearly, these extended latency periods may have implications for travelers, making genetic surveillance a crucial tool for the diagnosis, surveillance, and management of *P. ovale* malaria. In addition, it is recommended that microscopic techniques be combined with molecular methods (e.g., multiplex PCR) for accurate and specific diagnosis.

## CONCLUSION

In conclusion, all the information reported above highlights the importance of including specific primers not only for *P. ovale curtisi* but also for *P. ovale wallikeri* in the molecular diagnostic strategy for *Plasmodium* species.
